# Non-Lethal Heat Shock of the Asian Green Mussel, *Perna viridis*, Promotes Hsp70 Synthesis, Induces Thermotolerance and Protects Against Vibrio Infection

**DOI:** 10.1371/journal.pone.0135603

**Published:** 2015-08-19

**Authors:** Nor Afiqah Aleng, Yeong Yik Sung, Thomas H. MacRae, Mohd Effendy Abd Wahid

**Affiliations:** 1 Institute of Marine Biotechnology, Universiti Malaysia Terengganu (UMT), 21030, Kuala Terengganu, Malaysia; 2 School of Fisheries and Aquaculture Sciences, Universiti Malaysia Terengganu (UMT), 21030, Kuala Terengganu, Malaysia; 3 Department of Biology, Dalhousie University, Halifax, NS, B3H 4R2, Canada; Washington State University, UNITED STATES

## Abstract

Mild heat stress promotes thermotolerance and protection against several different stresses in aquatic animals, consequences correlated with the accumulation of heat shock protein 70 (Hsp70). The purpose of this study was to determine if non-lethal heat shock (NLHS) of the Asian green mussel, *Perna viridis*, an aquatic species of commercial value, promoted the production of Hsp70 and enhanced its resistance to stresses. Initially, the LT_50_ and LHT for *P*. *viridis* were determined to be 42°C and 44°C, respectively, with no heat shock induced death of mussels at 40°C or less. Immunoprobing of western blots revealed augmentation of constitutive (*Pv*Hsp70-1) and inducible (*Pv*Hsp70-2) Hsp70 in tissue from adductor muscle, foot, gill and mantel of *P*. *viridis* exposed to 38°C for 30 min followed by 6 h recovery, NLHS conditions for this organism. Characterization by liquid chromatography-tandem mass spectrometry (LC-MS/MS) revealed that *Pv*Hsp70-1 and *Pv*Hsp70-2 respectively corresponded most closely to Hsp70 from *P*. *viridis* and *Mytilus galloprovincialis*. Priming of adult mussels with NLHS promoted thermotolerance and increased resistance to *V*. *alginolyticus*. The induction of Hsp70 in parallel with enhanced thermotolerance and improved protection against *V*. *alginolyticus*, suggests Hsp70 functions in *P*. *viridis* as a molecular chaperone and as a stimulator of the immune system.

## Introduction

Aquatic organisms experience environmental stresses including temperature fluctuation, salinity shift, oxygen deprivation and pollution [1–3] as well as disease-causing biotic stressors such as bacteria, virus, fungi and parasites [4]. Stress disrupts the normal physiology and cellular homeostasis of all organisms, potentially resulting in their death [1, 5]. The heat shock response, an integral part of the physiological system that protects against environmental perturbations [6], involves the synthesis of heat shock proteins (Hsps) which, by molecular chaperone activity facilitate the proper folding of nascent proteins, prevent stress-induced irreversible protein denaturation and mediate storage and refolding of partially denatured protein [4]. Hsps also appear to stimulate the innate immune response of aquatic organisms thereby shielding cells against injury due to pathogens and making them more tolerant of disease and infection [7].

Non-lethal heat shock (NLHS) is an effective method to protect aquatic organisms against stress, an outcome often associated with increased Hsp accumulation [7, 5]. NLHS increases Hsp70 in the common carp, *Cyprinus carpio*, allowing it to survive a normally lethal temperature [8]. NLHS of *Artemia franciscana* promotes Hsp70 build-up, induces thermotolerance and guards *Artemia* larvae against *V*. *campbellii* and *V*. *proteolyticus*, two pathogens of this branchiopod crustacean [9, 10]. Exposing *Penaeus monodon* to a short hyperthermic stress enhances Hsp70 accumulation and resistance against gill associated virus (GAV) [11]. The concurrent induction of heat tolerance, resistance to bacterial infection and Hsp70 synthesis, suggests a role for Hsp70 in mediating the effects of stress perhaps via chaperoning and/or immune activation [12, 1, 4].

Several issues impede sustainable production of the Asian green mussel *Perna viridis* [13], a major aquaculture species in Malaysia, with fluctuation in water temperature due to climate change the most serious [14–17]. Additionally, bacteria, parasites and heavy metals hinder the successful cultivation of *P*. *viridis* and other bivalves in cage culture systems [18–20]. These types of problems occur in Marudu Bay, Malaysia, where temperature changes due to an influx of water lead to secondary infections by *V*. *alginolyticus*, a common pathogen of bivalves and crustaceans, causing mortalities of 95–98% in cultured *P*. *viridis*. Oysters cultivated in the same rafting area exhibit clinical signs similar to those of *P*. *viridis*. Sessile organisms like *P*. *viridis* depend on physiological responses such as the increased synthesis of Hsps to accommodate stresses because they cannot escape by swimming [1, 21]. Thus, the synthesis of Hsp70 in *P*. *viridis* upon NLHS was investigated in this study, revealing a potential role for this protein in tolerance to heat and resistance to bacterial infection.

## Materials and Methods

### Culture of *P*. *viridis*

Adult *P*. *viridis* measuring 70–80 mm in length were purchased from various long-line culture farms in Masai, Johor (1°29′36.5″N 103°52′40.94″E). Animals were acclimatized in the Universiti Malaysia Terengganu Marine Hatchery under constant aeration (>6 ppt) at 28°C and salinity of 30 ppt for two weeks prior to use. During acclimation, mussels were fed daily with the microalgae *Chaetoceros sp*. at 6 x 10^7^ cells/ml, the final number of algae in the tank. The rearing water was replaced every 2 days.

### Determination of Median Lethal Temperature (LT_50_), and Lethal Heat Temperature (LHT) for *L*. *viridis*

To determine the minimum temperatures that caused 50% mortality (LT50) and 100% mortality (LHT) groups of 20 *L*. *viridis* acclimatized at 28°C were exposed to abrupt 30 min heat shocks ranging in temperature from 34°C to 44°C in a water bath accurate to ±0.5°C. Mussels were then transferred to 28°C and mortality was determined 24 h later by counting live animals; gaping mussels that failed to respond to gentle tapping on the shell were considered dead. The percent mortality was calculated as (N_0_ - N_t_)/ N_0_ × 100 where N_0_ and N_t_ are initial and final numbers of living mussels [8]. Ten mussels were tested at each temperature and experiments were done in triplicate with non-heated animals as controls.

### Protein Extraction, SDS Polyacrylamide Gel Electrophoresis and Immunoprobing of Western Blots

For protein extraction approximately 100 mg of tissue prepared individually from the adductor muscle, foot, gill and mantle was rinsed with sterile, cold, distilled water several times, and homogenized in 500 μl cold buffer K (150 mM sorbitol, 70 mM potassium gluconate, 5 mM MgCl_2_, 5 mM NaH_2_PO_4_, 40 mM HEPES, pH 7.4) [22] containing a protease inhibitor cocktail (Sigma-Aldrich Inc, USA) [5]. Two-times concentrated SDS polyacrylamide gel electrophoreses sample buffer [23] was added to equal volumes of tissue homogenate, mixed by vortexing, heated at 95°C for 5 min, cooled and centrifuged at 2200 x g for 60 sec. Ten μl samples of the supernatant containing 0.2 mg protein were loaded in individual lanes of 7% SDS polyacrylamide gels and resolved by electrophoresis at 120 V for 15 min, followed by 150 V for 45 min. Two gels were run simultaneously of which one was stained with Biosafe Coomassie (BioRad Laboratories, USA) and the other blotted to polyvinylidene fluoride transfer membrane (BioRad Immun-Blot PVDF, USA). Membranes were incubated in 50 ml of blocking buffer which consisted of phosphate buffered saline containing 0.2% (v/v) Tween-20 and 5% (w/v) bovine serum albumin. Blots were then incubated for 60 min at room temperature with a mouse monoclonal antibody (Thermo Scientific, USA, MA3-006) diluted 1:5000 in PBS (BioRad Laboratories, USA) and which recognized both constitutive and inducible Hsp70. Goat anti-mouse IgG coupled with horseradish peroxidase (HRP) conjugate (Affinity BioReagents Inc., Golden, CO) was employed at a dilution of 1:5000 in PBS as secondary antibody. Diaminobenzidinetetrahydrocloride dehydrate (DAB) at 0.7 mM was used in association with 0.1% (v/v) H_2_O_2_ in 0.1 M Tris-HCl, pH 7.6, for detection of antibody reactive proteins [22, 10]. Human recombinant Hsp70 (Sigma Aldrich Inc.-H7283, USA) served as control for antibody reactivity. The blots were scanned with a GS-800 calibrated densitometer (BioRad Laboratories, USA) and quantification was performed by measuring the bands for *Pv*Hsp70-1 and *Pv*Hsp70-2 with Quantity One software (BioRad Laboratories, USA). The amounts of Hsp70 in tissues of *P*. *viridis* exposed to NLHS were interpreted as reflective density/mm^2^, the density value generated by Quantity One software. Human Hsp70 served as the control for antibody specificity.

### Determination of NLHS

Adult mussels (n = 5) acclimated at 28°C were abruptly heat shocked at temperatures ranging from 30 to 40°C for 30 min and then transferred to 28°C for 6 h recovery prior to protein extraction. Mussels held at 28°C served as controls. As determined by SDS polyacrylamide gel electrophoresis and immunoprobing of western blots the temperature that induced maximum Hsp70 accumulation in mussel tissues and did not result in mussel mortality was 38°C. In subsequent experiments, adult mussels (n = 30) acclimated to 28°C were heated at 38°C and allowed to recover at 28°C for 0, 6, 12, 24 and 48 h prior to protein extraction. After 48 h, protein was extracted every 2 days until 10 days post-heat shock. Determination of Hsp70 band density on Western blots, heat shock parameters and recovery conditions established that the optimal conditions for NLHS of *P*. *viridis* were 30 min heat shock at 38°C followed by 6 h recovery at 28°C, a value used in subsequent stress experiments.

### Identification of Hsp70 by Mass Spectrometry

In preparation for liquid chromatography-tandem mass spectrometry (LC-MS/MS) (Wischgoll et al., 2009), protein samples from mussels receiving NLHS were resolved in 5% SDS polyacrylamide gels by electrophoresis, stained with Biosafe Coomassie (BioRad Laboratories) and destained. Gel slices containing *Pv*Hsp70-1 and *Pv*Hsp70-2 were excised and freeze-dried overnight. Proteins were then digested with trypsin and the extracted peptides [24] were loaded onto a C18 column 300SB, 3.5 μm column (Agilent Technologies, USA) and separated with a linear gradient of water/acetonitrile/0.1% formic acid (v/v). The peptides were analyzed by electrospray ionization mass spectrometry with a Shimadzu Prominence Nano HPLC system (Shimadzu, Japan) coupled to a 5600 TripleTOF mass spectrometer (AB Sciex, USA). Protein identification was performed with Mascot sequence matching software (Matrix Science, USA) and the Ludwig NR database.

### NLHS and the Induction of Heat and Bacterial Tolerance in *P*. *viridis*

Thermotolerance induction was determined by challenging mussels exposed to NLHS at their LT50 and LHT with survival ascertained 24 h later as described above. Ten mussels were used for each treatment and experiments were done in triplicate. Control animals were held at 28°C.

To determine LC50, the concentration of *V*. *alginolyticus* causing 50% mortality of mussels, bacteria were grown overnight at 28°C with constant shaking in marine nutrient broth to stationary phase, harvested and suspended in sterile seawater prior to determining density at 600 nm. The number of bacteria was calculated from a standard curve obtained according to the equation, y = (2 x 10^8^)x – (3 x 10^7^), where y is the number of bacteria/ml and x is the OD_600_ value. Mussels were incubated by immersion with 1 x 10^6^, 1 x 10^7^, 1 x 10^8^ and 1 x 10^9^ *V*. *alginolyticus*/ml for various times. Survival was determined daily by counting live animals. Gaping mussels that failed to respond to gentle tapping on the shell were considered dead. The experiment was done in triplicate.

Mussels (n = 10) subjected to NLHS were challenged with 1 x 10^8^ *V*. *alginolyticus*/ml, for 72 h, the LC_50_, after which survival was determined as described in the previous section. Mussels challenged with *V*. *alginolyticus* without NLHS served as controls. The experiment was done in triplicate.

### Data Analysis

Survival percentages were ArcSin-transformed to satisfy normality. The significance of differences between survival of groups of challenged mussels either exposed or not exposed to NLHS and the amounts of Hsp70 were evaluated by using one way ANOVA with SPSS version 20.0 for Windows.

## Results

### LT_50_ and LHT for Adult *P*. *viridis*

**Fig 1:**
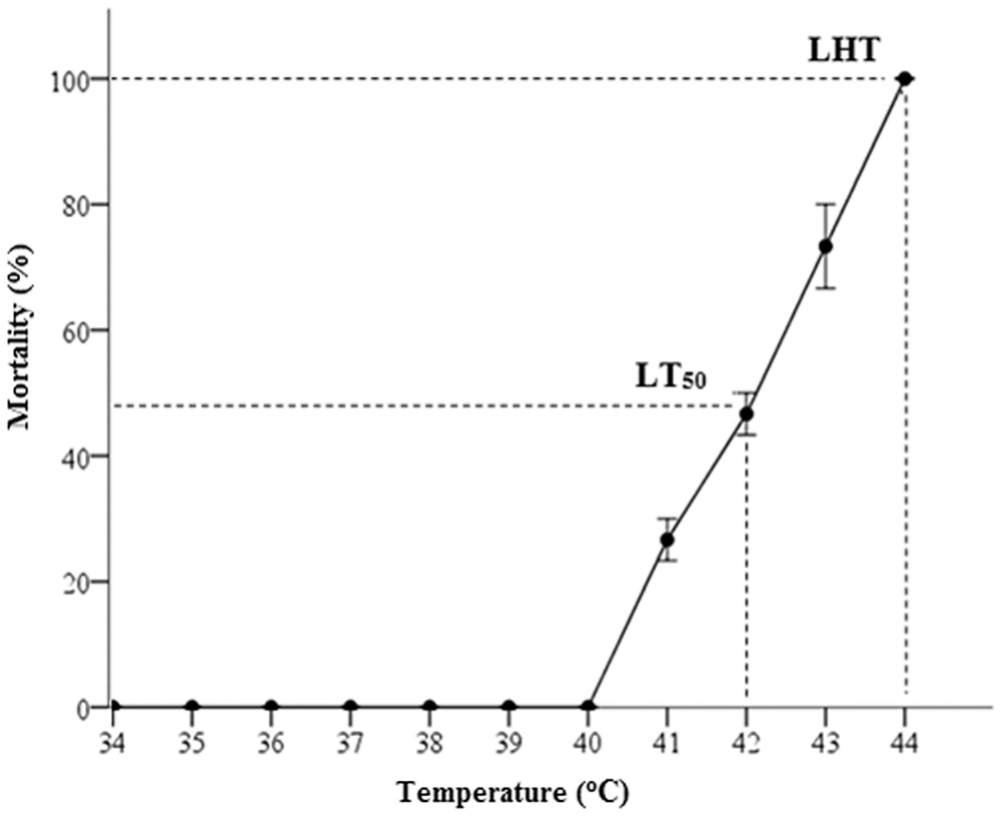
LT_50_ and LHT for *P*. *viridis*. The mortality of *P*. *viridis* adults acclimatized at 28°C and heat shocked for 30 min at 1°C intervals was determined as described in Methodology. LT_50_, median lethal heat treatment; LHT, lethal heat treatment. Experiments were done in triplicate. Data are presented as mean ± standard errors.

Heating for 30 min at temperatures ranging from 34°C to 40°C did not kill adult *P*. *viridis* but mortality occurred above 40°C, with LT_50_ and LHT at 42°C and 44°C respectively (Fig 1).

### Heat Shock Induced Hsp70 in *P*. *viridis*

**Fig 2:**
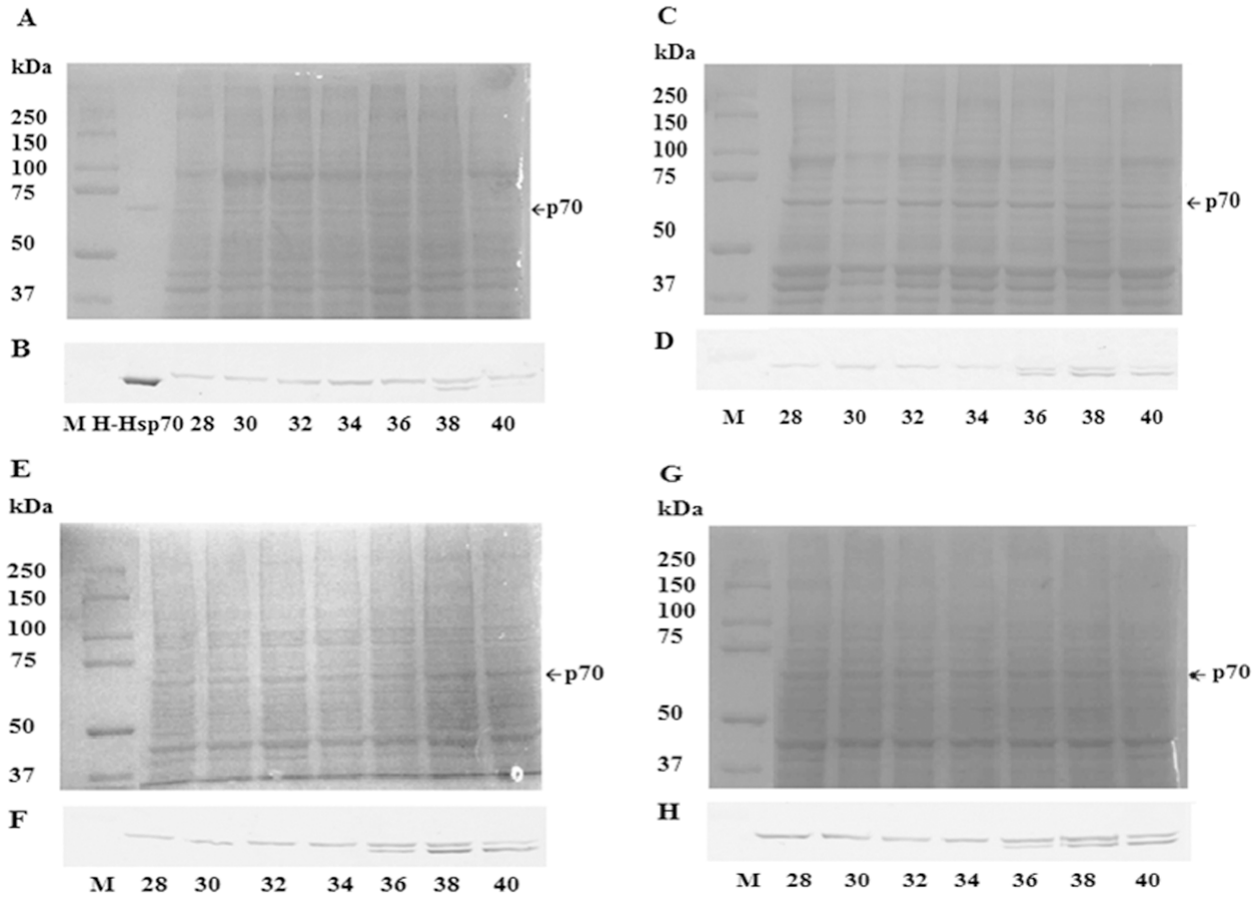
Hsp70 was induced in *P*. *viridis* by heat shock. Protein samples from the adductor muscle (A, B), foot (C, D), gill (E, F) and mantel (G, H) were resolved in 7% SDS polyacrylamide gels and either stained with Coomassie blue (A, C, E, G) or blotted to membranes and probed with antibody to Hsp70 (B, D, F, H). Arrows labeled p70 indicate the position of 70 kDa proteins in the gel. M, molecular mass markers in kDa; H-Hsp70, recombinant human Hsp70.

**Fig 3:**
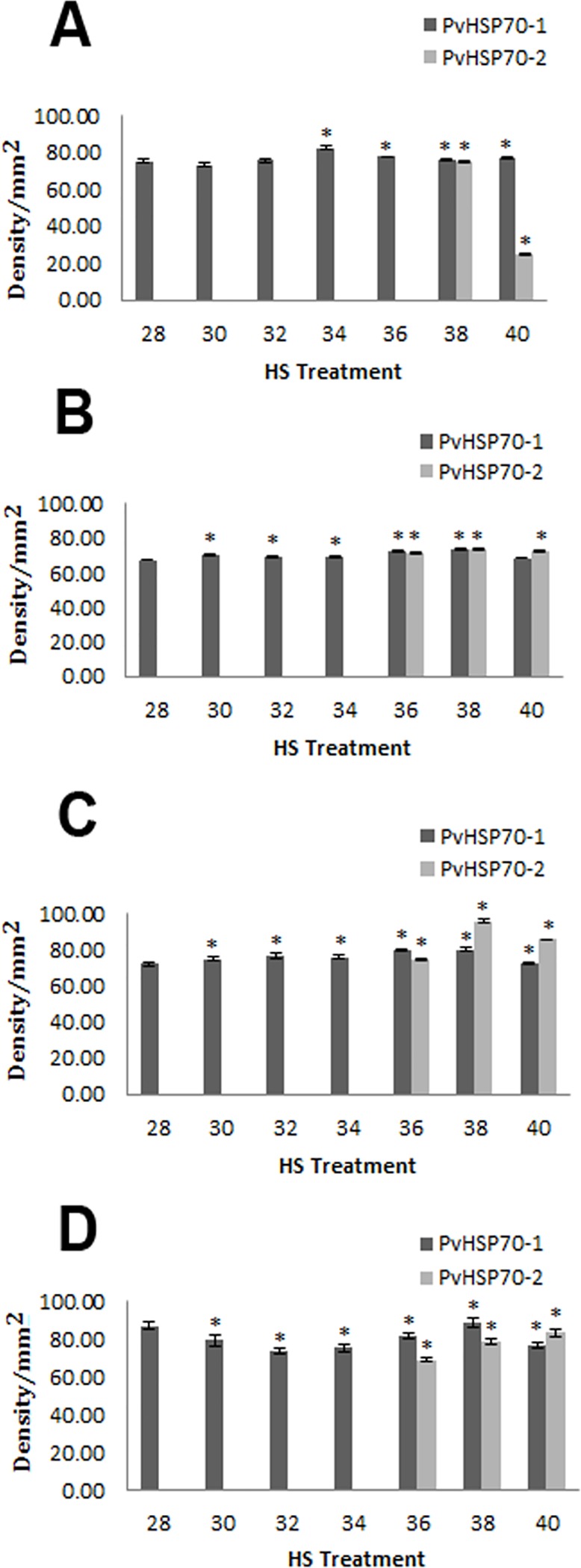
NLHS induced the synthesis of Hsp70 in *P*. *viridis*. The amounts of *Pv*Hsp70-1 and *Pv*Hsp70-2 in (A) adductor muscle, (B) foot, (C) gill and (D) mantle of *P*. *viridis* exposed to heat shock at 30, 32, 34, 36, 38 and 40°C were determined by densitometry analysis of antibody-stained Western blots as described in Materials and Methods. Bars that are not visible denote the absence of *Pv*Hsp70-2. Asterisk (*) represents statistical difference against the control treatment (*P*<0.05). The experiment was performed in duplicate. 28, mussels not receiving NLHS (control).

Following 6 h recovery 70 kDa proteins were observed when extracts of adductor muscle, foot, gill and mantle from *P*. *viridis* heated at temperatures from 28–40°C were resolved in 7% SDS polyacrylamide gels and stained with Coomassie blue (Figs 2 and 3). Immunoprobing of polyvinylidene fluoride membranes containing protein extracts resolved in SDS polyacrylamide gels with a monoclonal antibody to Hsp70 revealed single 70 kDa bands at lower temperatures but two bands at higher temperatures (Fig 2). The upper band was termed *Pv*Hsp70-1 and the lower *Pv*Hsp70-2. The amount of *Pv*Hsp70-1 visible in immunostained blots increased at 34 and 36°C in adductor tissue and at 36 and 38°C in other tissues (S1 Table) (S1A Table). *Pv*Hsp70-2 was induced at 38 and slightly at 40°C in adductor muscle, whereas in foot, gill and mantle *Pv*Hsp70-2 was apparent at 36, 38 and 40°C (S1B Table). Heat shock at 40°C all but eliminated *Pv*Hsp70-2 in adductor muscle and reduced *Pv*Hsp70-1 in all organs with adductor muscle also showing a decline at 38°C. *Pv*Hsp70-1 and *Pv*Hsp70-2 protein bands on Western blots were scanned, revealing bands that were significantly different from control values (Fig 3). In the following experiments 38°C was used for heat shock because the synthesis of *Pv*Hsp70-1 and *Pv*Hsp70-2 was enhanced at this temperature in all tissues of *P*. *viridis* examined and no death of mussels occurred.

### *P*. *viridis* Hsp70 Varied with Recovery Time after Heat Shock

**Fig 4:**
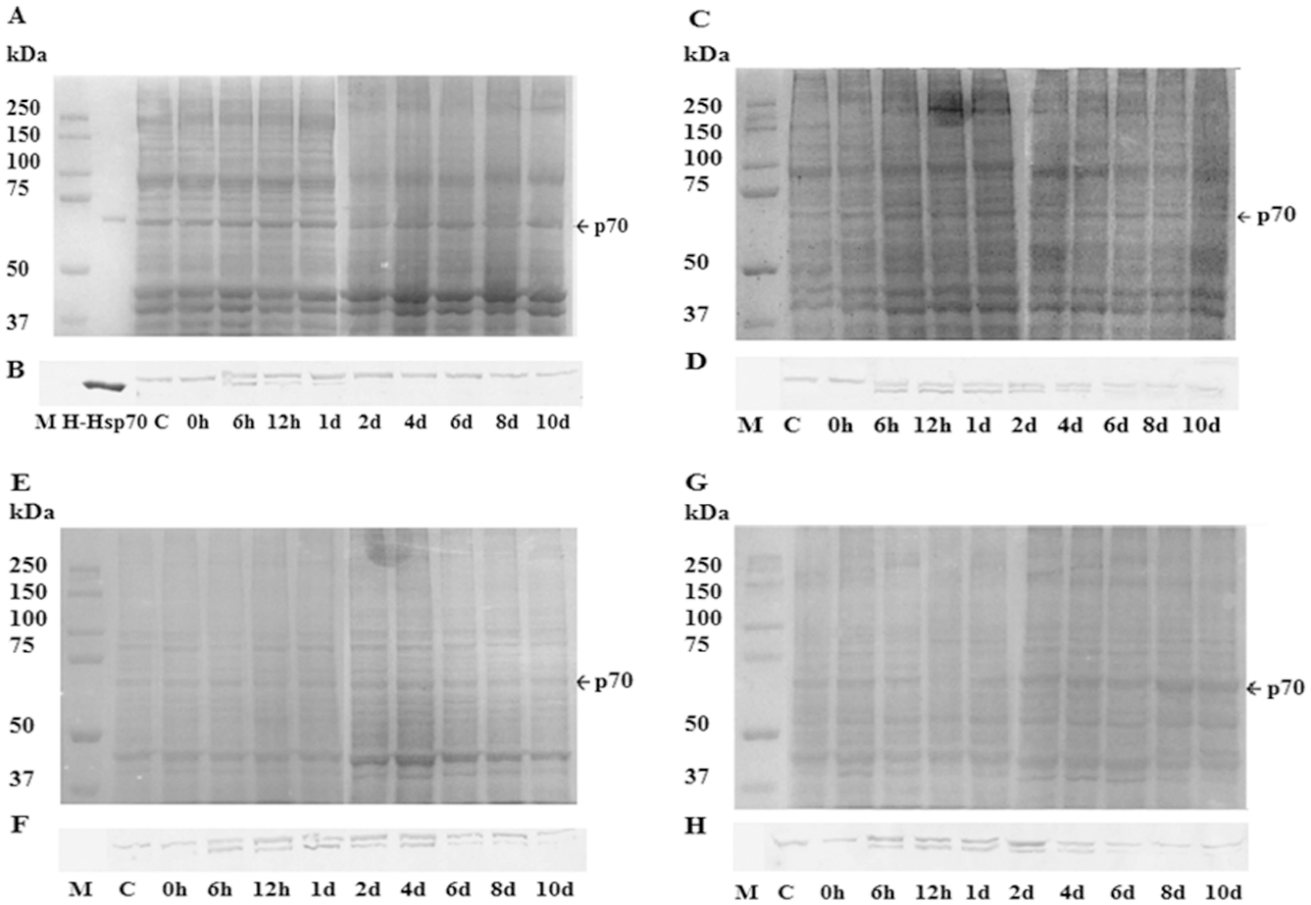
*P*. *viridis* Hsp70 Varied with Recovery Time after Heat Shock. Protein samples from the adductor muscle (A, B), foot (C, D), gill (E, F) and mantel (G, H) were resolved in 7% SDS polyacrylamide gels and either stained with Coomassie blue (A, C, E, G) or blotted to membranes and probed with antibody to Hsp70 (B, D, F, H). Arrows labeled p70 indicate the position of 70 kDa proteins in the gel. M, molecular mass markers in kDa; H-Hsp70, recombinant human Hsp70. C, mussels not receiving heat shock.

**Fig 5:**
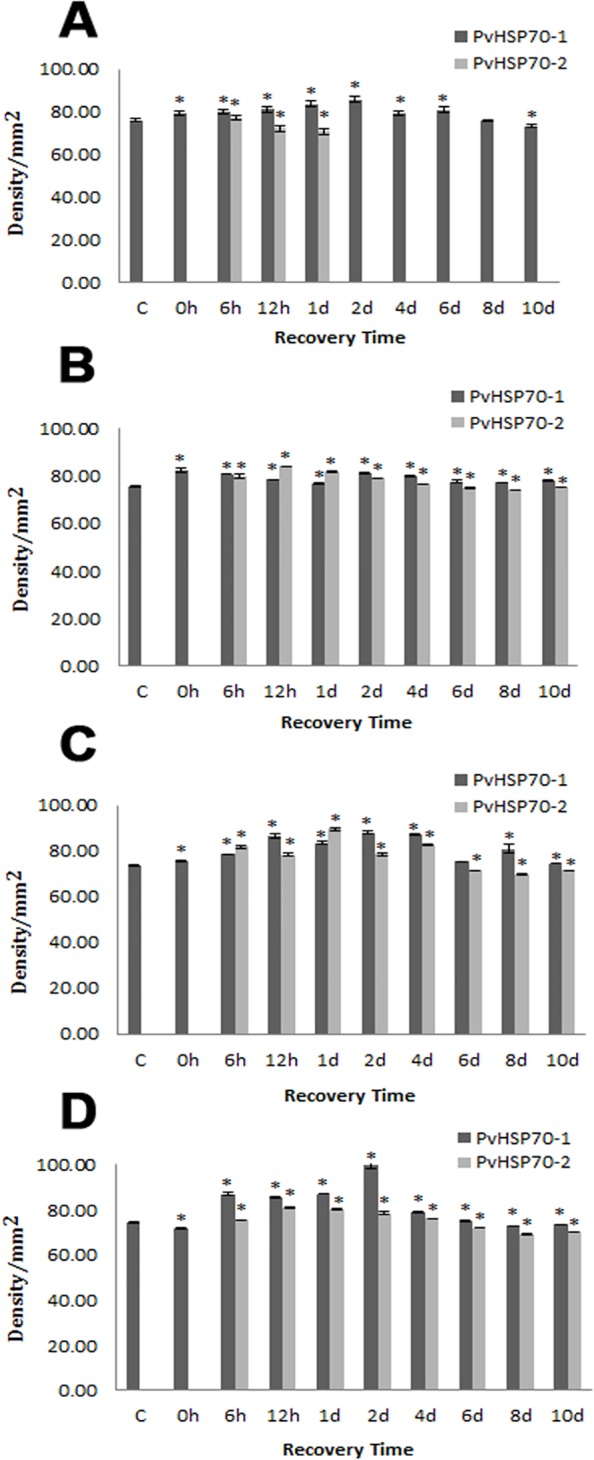
*P*. *viridis* Hsp70 Varied with Recovery Time after Heat Shock. The amounts of *Pv*Hsp70-1 and *Pv*Hsp70-2 in the (A) adductor muscle, (B) foot, (C) gill and (D) mantle of *P*. *viridis* upon NLHS at 38°C with different recovery length were determined as just described. Data are presented as mean ± standard errors. Bars that are not visible denote the absence of *Pv*Hsp70-2. Asterisk (*) represents statistical difference against the control treatment (*P*<0.05). The experiment was performed in duplicate. c and 28, mussels not receiving NLHS (control).

*Pv*Hsp70-2 was induced in all tissues examined when *P*. *viridis* were heated at 38°C for 30 min followed by 6 h recovery (Figs 2, 4 and 5) (S1D Table). In adductor muscle, *Pv*Hsp70-2 declined at 12 h and was not detectable at day 2 whereas *Pv*Hsp70-1 was reduced at day 8 but still visible at day 12 (S1C Table). In foot, gill and mantle, *Pv*Hsp70-2 was present at day 4 but was difficult to detect at day 6. *Pv*Hsp70-1 decreased at either day 4 or 6 in these three tissues (Figs 4 and 5). Heat shock at 38°C for 30 min with 6 h recovery induced *Pv*Hsp70-2 in all tissues of *P*. *viridis* examined, hence these conditions were used for NLHS in subsequent experiments.

### Identification of Hsp70 Isotypes in *P*. *viridis* by Mass Spectrometry

**Fig 6:**
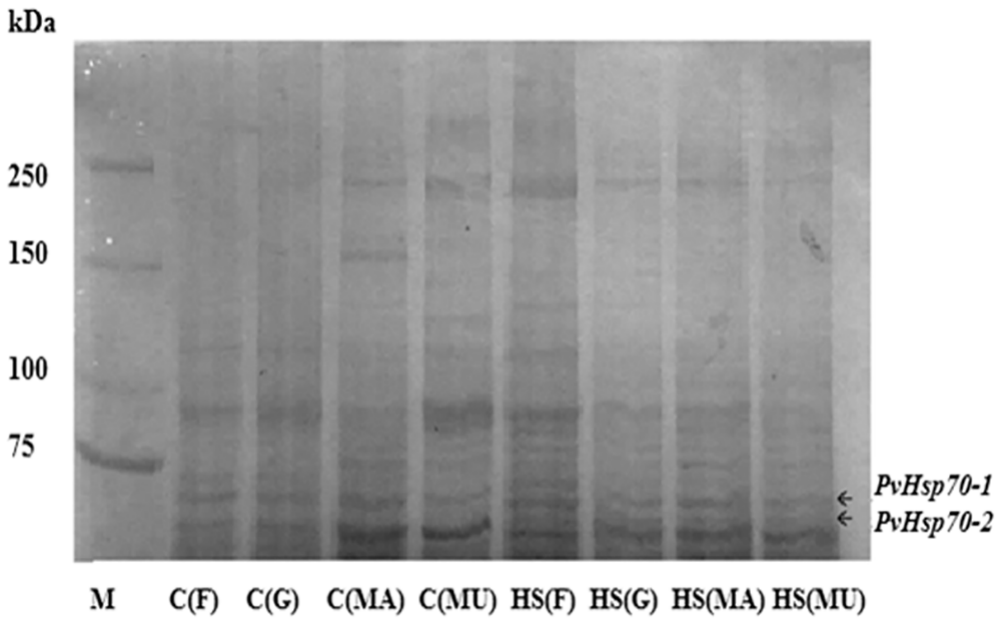
Two 70 kDa protein bands were resolved in 5% SDS polyacrylamide gels. Tissues corresponding to the adductor muscle, foot, gill and mantel were isolated from *P*. *viridis* exposed to NLHS, homogenized, centrifuged and applied to a 5% SDS polyacrylamide gel. Labeled arrows indicate excised regions of the gel subsequently shown by mass spectrometry to contain *Pv*Hsp70-1 and *Pv*Hsp70-2. M, molecular mass in kDa; C, mussels not receiving NLHS; HS, mussels receiving NLHS; F, foot; G, gill; MA, mantle; MU, adductor muscle.

Although only a single prominent band was distinguished around 70 kDa in 7% SDS polyacrylamide gels (Fig 2), two bands were seen upon electrophoresis in 5% gels (Fig 6). The two bands were excised and resident proteins analyzed by mass spectrometry. The upper band contained an Hsp70 equivalent to *Pv*Hsp70-1 and the lower band an Hsp70 corresponding to *Pv*Hsp70-2. Utilization of Mascot sequence matching software with Ludwig NR database revealed that *Pv*Hsp70-1 and *Pv*Hsp70-2 were respectively most similar to Hsp70 from *P*. *viridis* and *M*. *galloprovincialis* (Table 1). Both isotypes were similar to Hsp70s in other bivalves (Table 2).

**Table 1:** Identification of *Pv*Hsp70-1 and *Pv*Hsp70-2 by LC-MS/MS.

Protein^a^	Peptide Sequence ^b^	GenBank Accession No.^c^	Identified Protein Name^d^	Coverage (%)^e^	MASCOT Score^f^
*Pv*Hsp70-1	R.LSKEEIER.M	ABJ98722	Hsp71 [*Perna viridis*]	34%	1602
	K.ITITNDKGR.L				
	K.STVEDEKLK.D				
	K.VEIIANDQGNR.T				
	K.DAGTISGMNVLR.I				
	K.MKETAESYLGK.T				
	K.NSLESYAFNMK.S				
	R.KFDDASVQSDMK.H				
	K.ASIHDIVLVGGSTR.I				
	R.MVNHFIQEFKR.K				
	R.ARFEELNADLFR.G				
	K.TFFPEEISSMVLVK.M				
	R.IINEPTAAAIAYGLDK.K				
	K.NQVAMNPVNTVFDAK.R				
	K.STSGDTHLGGEDFDNR.M				
	R.IINEPTAAAIAYGLDKK.A				
	K.MDKASIHDIVLVGGSTR.I				
	K.NQVAMNPVNTVFDAKR.L				
	K.TITNSVVTVPAYFNDSQR.Q				
*Pv*Hsp70-2	R.LSKEDIDR.M	CAH04107	Hsp70 [*Mytilus galloprovincialis*]	16%	575
	K.FDLTGIPPAPR.G				
	R.NQLENYIFSVK.Q				
	K.MKETAEAYLGQK.V				
	R.IINEPTAAALAYGLDK.N				
	R.STAGDTHLGGEDFDNR.M				

Mass spectra were analyzed to identify proteins of interest using Mascot sequence matching software with the Ludwig NR database. Peptides were assessed individually with a score greater than 44 considered significant. Data shown for both proteins are the highest scores matched.

^a^Proteins identified in excised gel slices.

^b^Peptide sequences derived from gel samples.

^c^Accession numbers for proteins generated by database searching.

^d^Identified protein name, best match with the Hsp70s identified from the gels.

^e^Coverage (%), Sequence coverage for the most closely matched protein.

^f^Combined scores of all observed mass spectra that matched the amino acid sequences within the protein of interest.

**Table 2:** Comparison of *Pv*Hsp70-1 and *Pv*Hsp70-2 with Hsp70 from bivalves.

Protein	Ludwig NR Accession No^a^	GenBank Accession No.	IdentifiedProtein Names	Nominal Mass (Da)^b^	pI Value^c^	Coverage (%)	MASCOT Scores
***Pv*HSp70-1**	A5Y8F9	ABJ98722	Hsp71 [*Perna viridis*]	71467	5.25	34	1602
	Q3LF65	CAH04109	Hsc71 [*Mytilus galloprovincialis*]	71280	5.29	22	1033
	H3JZA0	BAL52328	Hsp70 [*Pinctada fucata*]	71446	5.31	18	777
	A2TF45	ABM92345	Hsp70 [*Laternula elliptica*]	71222	5.20	19	758
	K4FC97	AFH66950	Hsp70 [*Tegillarca granosa*]	71438	5.32	18	742
	Q6XVG4	AAO38780	Hsp70 [*Azumapecten farreri*]	71217	5.33	18	710
	L0BUQ9	AFZ93094	Hsp70 [*Paphia undulata*]	71207	5.51	18	689
	F8RUS3	ADT78476	Hsp70 [*Meretrix meretrix*]	71390	5.32	15	646
	Q8WQ17	CAC83684	Hsc70 [*Ostrea edulis*]	64836	5.51	13	513
***Pv*Hsp70-2**	Q3LF67	CAH04107	Hsp70 [*Mytilus galloprovincialis*]	69563	5.30	16	575
	F8RUS3	ADT78476	Hsp70 [*Meretrix meretrix*]	71390	5.32	5	223

A higher score indicates a better match.

^a^Accession number generated by use of Mascot search and the Ludwig NR database.

^b^Mass of identified proteins.

^c^Isoeletric point.

### NLHS Promoted Thermotolerance in *P*. *viridis*

**Fig 7:**
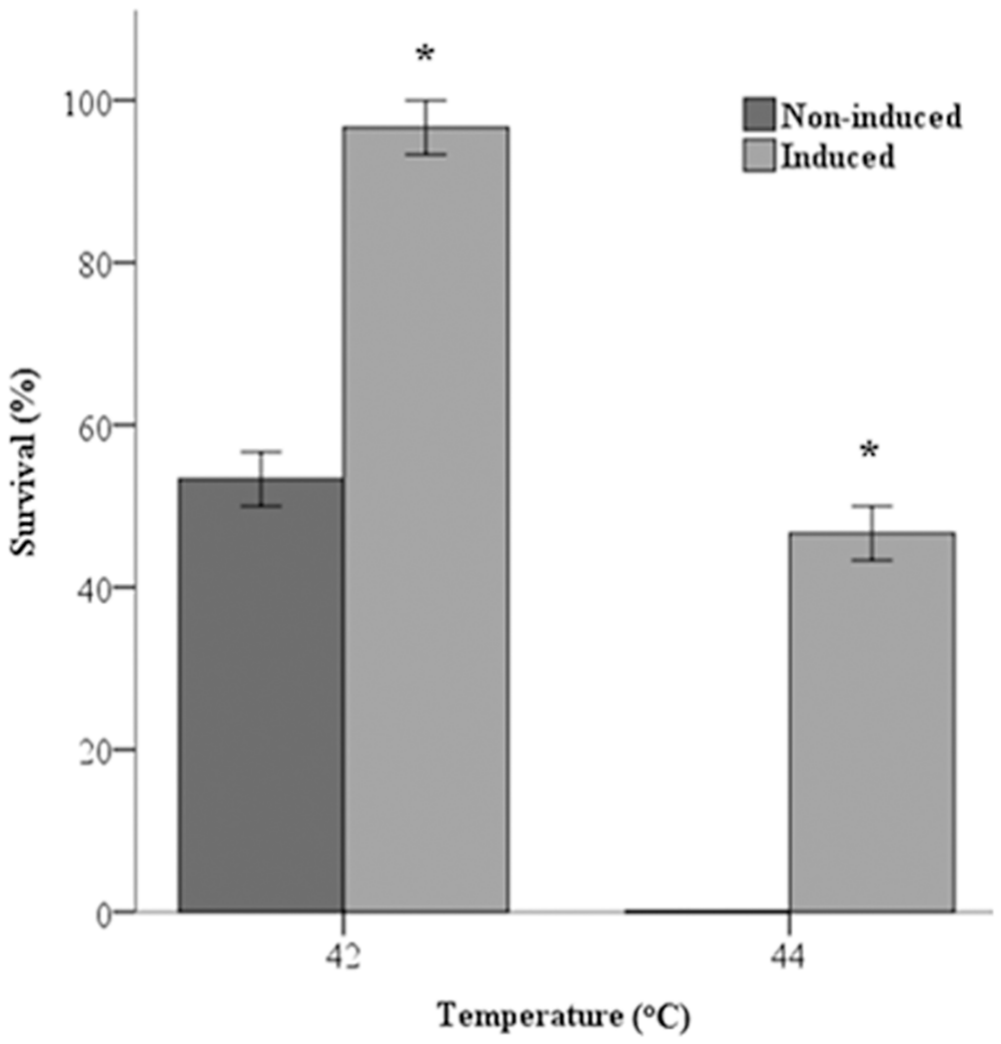
NLHS enhanced the thermotolerance of *P*. *viridis*. *P*. *viridis* acclimatized at 28°C were exposed to NLHS and then heated for 30 min at their LT_50_ (42°C) and LHT (44°C). Survivors were counted 24 h after challenge. Data are presented as mean ± standard errors. Asterisk (*) represents statistical difference against the control (*P*< 0.05). The experiments were performed in triplicate. Non-induced, mussels not receiving NLHS; Induced, mussels exposed to NLHS.

Exposure to NLHS increased the survival of mussels approximately 2-fold upon LT50 challenge whereas 50% of animals primed with NLHS were viable after LHT (Fig 7).

### NLHS Increased the Tolerance of *P*. *viridis* to Bacterial Infection

**Fig 8:**
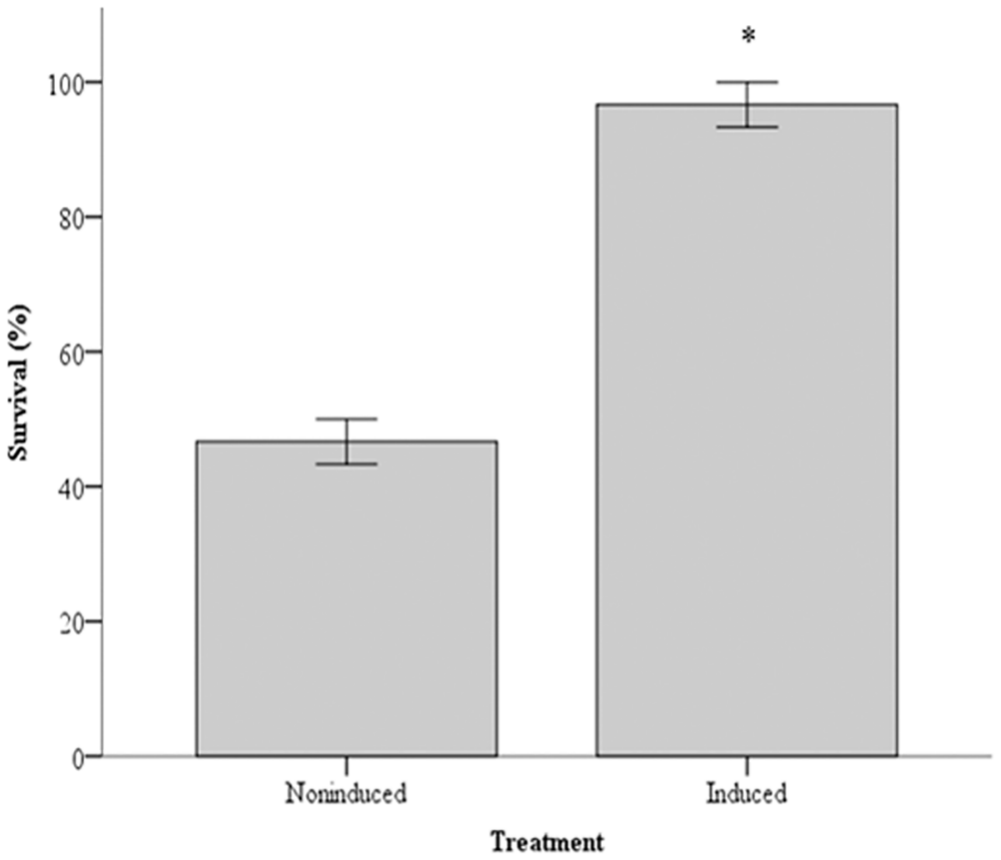
NLHS increased the bacterial tolerance of *P*. *viridis*. *P*. *viridis* acclimatized at 28°C were exposed to NLHS, and then incubated with 1 x 10^8^ *V*. *alginolyticus*/ml, the LC_50_ for this species. Survivors were counted 72 h after challenge. Data are presented as mean ± standard errors. Asterisk (*) represents statistical difference against the control (*P*< 0.05). The experiment was performed in triplicate. Non-induced, mussels not receiving NLHS; Induced, mussels exposed to NLHS.

Exposure of non-heated *P*. *viridis* to *V*. *alginolyticus* for 72 h revealed an LC_50_ of 1.0 x 10^8^ bacteria/ml (Table 3). Priming *P*. *viridis* with NLHS enhanced survival upon 72 h exposure to 1 x 10^8^ *V*. *alginolyticus*/ml from 50% to almost 100% (Fig 8).

**Table 3:** Survival of *P*. *viridis* adults after exposure to *V*. *alginolyticus*.

*V*. *alginolyticus*	Survival (%)		
	24 h	48 h	72 h
0	100	100	100
1 x 10^6^	100	100	100
1 x 10^7^	100	100	97±3
1 x 10^8^	100	93±3	50±3*
1 x 10^9^	100±3	70±6*	0*

The survival of *P*. *viridis* was determined after exposure to various concentrations of *V*. *alginolyticus* for the times indicated. The standard error was determined for each survival value (mean ± SE). The experiment was done in triplicate. Values with asterisk (*) are significantly different (P< 0.05).

## Discussion

The synthesis of Hsp70, the best studied stress protein, is induced in aquatic animals by heat stress [25, 4, 26] and in this work immunoprobing of western blots revealed that two Hsp70 isotypes increased in *P*. *viridis* after NLHS. The *P viridis* Hsp70s, *Pv*Hsp70-1 and *Pv*Hsp70-2, respectively matched Hsp70 in *P*. *viridis* and *M*. *galloprovicialis* most closely, while being similar to Hsp70s from other bivalves. *Pv*Hsp70-1 and PvHsp70-2 increased in the adductor muscle, foot, gill and mantle of *P*. *viridis* upon NLHS. Accumulation of Hsp70 in the tissues examined in this study was also seen in *O*. *edulis*, *M*. *galloprovincialis*, and *C*. *gigas* [22, 27, 28].

*Pv*Hsp70-1 was observed in mussel tissues before heat shock indicating that it was produced constitutively, whereas *Pv*Hsp70-2 was not apparent until recovery after NLHS at temperatures favourable for growth, observations similar to those for other aquatic organisms exposed to heat perturbation [9]. *Pv*Hsp70-2, the inducible isotype of Hsp70, was present in all tissues of *P*. *viridis* examined after 6 h recovery from heat shock, but shorter post-shock times were not examined so synthesis may have occurred earlier. *Pv*Hsp70-2 was induced maximally by a 30 min heat shock at 38 to 40°C, in line with findings that Hsp70 is induced when aquatic organisms experience temperatures 5–10°C above their normal growth requirement [29, 30]. *Pv*Hsp70-2 persisted for several hours after induction by NLHS perhaps providing protection against subsequent stress, but their synthesis eventually decreased as seen in other species [29]. Constitutive Hsp70, transiently induced by heating *P*. *viridis*, may function cooperatively with inducible Hsp70 to protect cells against heat, pathogens, heavy metals and other insults [31, 7, 4]. *P*. *viridis* Hsp70 may be crucial for cell survival during stress, expediting protein repair and reducing protein denaturation that could lead to death [32, 4]. In this study, a 30 min heat shock at 38°C with 6 h recovery was selected as the NLHS because this treatment increased the amount of *Pv*Hsp70-1 and *Pv*Hsp70-2 in *P*. *viridis* without causing mortality. The possibility of Hsp70 breakdown generating a second band is low because the mass-spectrometry indicated two different Hsp70s and protease inhibitors were used when making the protein extract.

Induced thermotolerance refers to the ability of an organism to withstand an otherwise lethal temperature, a condition achieved by priming animals with NLHS [8]. In this study, groups of mussels primed with NLHS and exhibiting increased Hsp70 survived LT50 challenge, whereas approximately 50% survived LHT challenge. Clearly, NLHS promoted thermotolerance in *P*. *viridis*, an observation similar to that for *Mytilus edulis* where exposure to a short heat shock increased resistance to lethal heat for 3 d [33]. In other bivalves, constitutively expressed and induced stress proteins are thought to mediate thermotolerance and this may require their cooperation. For example, the up-regulation of constitutive 77 and 72 kDa proteins and the synthesis of an inducible 69 kDa protein by NLHS promote thermotolerance in *C*. *gigas* [22]. Additionally, increases in constitutive and inducible Hsp70s correlate with thermotolerance induction in the adult oyster, *Ostreola conchaphila*, [34] and Hsp70 accumulation parallels increasing protection of *A*. *irradians irradians* juveniles against LHT, with thermotolerance lasting at least 7 days [35]. The correlation between Hsp70 accumulation and increasing thermotolerance in bivalves is observed for other aquatic organisms such as fish and shrimp [4]. These studies indicate the importance of constitutive and inducible Hsp70 isotypes in bivalve thermotolerance, however Hsps in addition to Hsp70 may contribute to thermotolerance by protecting proteins against heat denaturation and assisting protein refolding [36–38], both vital to cell homeostasis.

Cross-protection or cross-tolerance, an enhanced tolerance to a particular stress, acquired by an initial transient, but different, stress [39, 40, 7, 4], was demonstrated by challenging *P*. *viridis* subjected to NLHS with *V*. *alginolyticus*. The enhanced protection of *P*. *viridis* against *V*. *alginolyticus* correlated with increasing amounts of *Pv*Hsp70-1 and *Pv*Hsp70-2 in all tissues of *P*. *viridis* examined. A role for Hsp70 in averting infection is suggested for *C*. *virginica* where the augmentation of a constitutive 69 kDa protein and the induction of a 72 kDa protein after sub-lethal heat shock promotes survival upon challenge with *Perkinsus marinus* [41]. The accumulation of Hsp70 after a short heat stress corresponds to increased resistance of *P*. *monodon* against gill associated virus (GAV), an effect accompanied by reduction of viral replication [11]. Additionally, accumulation of Hsp70 after NLHS enhances the tolerance of *A*. *franciscana* larvae against pathogenic *V*. *campbellii* and *V*. *proteolyticus* (Sung et al., 2007). Clearly, the accumulation of Hsp70s induced by NLHS correlates with survival against subsequent infection, suggesting Hsp70 influences the immune response. Hsps may stabilize cells against injury in response to pathogen proliferation, mediate folding of cell proteins synthesized due to bacterial pathogens, store and re-fold partially denatured protein and stimulate the innate immune response, possibly by sending danger signals to the innate immune system [42–44, 4, 7].

Although bivalves rely solely on an innate, non-lymphoid system of immune responses [1, 45], some of the immune mechanisms in bivalves are structurally and functionally similar to those in vertebrates [46]. Hsp70 attenuates infections in vertebrates by activating toll-like receptors (TLRs) and transducing signals from inflammatory reactions to cells of the innate immune system such as macrophages, dendritic cells and neutrophils [47, 48]. The extracellular Hsp70 family promotes inflammatory cytokine production [49] and may elicit production of inducible nitric oxide synthase [50], interleukin (IL) 1-β, IL 6 and tumor necrocis factor α (TNFα) [51], to guard against infection. Invertebrate Hsps restrict bacterial infection by activation of TLRs [7], but there is no evidence indicating a relationship between Hsps and TLRs in bivalves. Considering that TLR genes occur in bivalves such as *C*. *farreri* [52], *C*. *virginica* [53], *A*. *irradians* [54] and *M*. *mercenaria* [55] immune activation via TLRs is possible in *P*. *viridis*.

The data presented herein demonstrate that Hsp70 is induced in *P*. *viridis* by NLHS and that Hsp70 plays a role in increasing the thermotolerance of *P*. *viridis* and enhancing survival against *V*. *alginolyticus* challenge. Further work is required to elucidate the role of Hsps in induced thermotolerance and the immune response of mussels, perhaps with the application of molecular tools such as RNA interference (RNAi) an option. Such studies are of fundamental interest and have applied significance through formulation of strategies to protect aquatic organisms against stress and disease, of particular importance in aquaculture.

## Supporting Information

S1 Table Amounts of *Pv*Hsp70-1 interpreted as reflective density/mm^2^ in tissues of *P*. *viridis* exposed to NLHS. The amounts of *Pv*Hsp70-1 in adductor muscle, foot, gill and mantle of *P*. *viridis* exposed to heat shock at 30, 32, 34, 36, 38 and 40°C were determined by densitometry analysis of antibody-stained Western blots as described in Materials and Methods. Data are presented as mean ± standard deviation. Asterisk (*) represents statistical difference against the control treatment (*P*<0.05). 28, mussels not receiving NLHS (control) (S1A Table). Amounts of *Pv*Hsp70-2 interpreted as reflective density/mm^2^ in tissues of *P*. *viridis* exposed to NLHS. The amounts of *Pv*Hsp70-2 in adductor muscle, foot, gill and mantle of *P*. *viridis* exposed to heat shock at 30, 32, 34, 36, 38 and 40°C were determined by densitometry analysis of antibody-stained Western blots as described in Materials and Methods. Data are presented as mean ± standard deviation. Asterisk (*) represents statistical difference against the control treatment (*P*<0.05). 28, mussels not receiving NLHS (control) (S1B Table). Amounts of *Pv*Hsp70-1 interpreted as reflective density/mm^2^ in tissues of *P*. *viridis* upon NLHS at 38°C with different recovery length. The amounts of *Pv*Hsp70-1 in the adductor muscle, foot, gill and mantle of *P*. *viridis* upon NLHS at 38°C with different recovery length. Data are presented as mean ± standard deviation. Asterisk (*) represents statistical difference against the control treatment (*P*<0.05). c and 28, mussels not receiving NLHS (control) (S1C Table). Amounts of *PvHsp70-2* interpreted as reflective density/mm^2^ in tissues of *P*. *viridis* upon NLHS at 38°C with different recovery length. The amounts of *Pv*Hsp70-1 in the adductor muscle, foot, gill and mantle of *P*. *viridis* upon NLHS at 38°C with different recovery length. Data are presented as mean ± standard deviation. Asterisk (*) represents statistical difference against the control treatment (*P*<0.05). c and 28, mussels not receiving NLHS (control) (S1D Table).
(DOCX)
