# Supplementary material for: Non-Lethal Heat Shock of the Asian Green Mussel, Perna viridis, Promotes Hsp70 Synthesis, Induces Thermotolerance and Protects Against Vibrio Infection
Source: PLoS One. 2015 Aug 19;10(8):e0135603. doi: 10.1371/journal.pone.0135603 (PMC4546054; doi:10.1371/journal.pone.0135603)
Supplement: S1 Table — The amounts of PvHsp70-1 in adductor muscle, foot, gill and mantle of P. viridis exposed to heat shock at 30, 32, 34, 36, 38 and 40°C were determined by densitometry analysis of antibody-stained Western blots as described in Materials and Methods. Data are presented as mean ± standard deviation. Asterisk (*) represents statistical difference against the control treatment (P<0.05). 28, mussels not receiving NLHS (control) (S1A Table). Amounts of PvHsp70-2 interpreted as reflective density/mm2 in tissues of P. viridis exposed to NLHS. The amounts of PvHsp70-2 in adductor muscle, foot, gill and mantle of P. viridis exposed to heat shock at 30, 32, 34, 36, 38 and 40°C were determined by densitometry analysis of antibody-stained Western blots as described in Materials and Methods. Data are presented as mean ± standard deviation. Asterisk (*) represents statistical difference against the control treatment (P<0.05). 28, mussels not receiving NLHS (control) (S1B Table). Amounts of PvHsp70-1 interpreted as reflective density/mm2 in tissues of P. viridis upon NLHS at 38°C with different recovery length. The amounts of PvHsp70-1 in the adductor muscle, foot, gill and mantle of P. viridis upon NLHS at 38°C with different recovery length. Data are presented as mean ± standard deviation. Asterisk (*) represents statistical difference against the control treatment (P<0.05). c and 28, mussels not receiving NLHS (control) (S1C Table). Amounts of PvHsp70-2 interpreted as reflective density/mm2 in tissues of P. viridis upon NLHS at 38°C with different recovery length. The amounts of PvHsp70-1 in the adductor muscle, foot, gill and mantle of P. viridis upon NLHS at 38°C with different recovery length. Data are presented as mean ± standard deviation. Asterisk (*) represents statistical difference against the control treatment (P<0.05). c and 28, mussels not receiving NLHS (control) (S1D Table). (DOCX) [file pone.0135603.s001.docx]

S1A Table.

| HS Treatment | *Pv*Hsp70-1 (mean density ± stdev/mm^2^) | | | |
| --- | --- | --- | --- | --- |
| (^o^C) | Adductor muscle | Foot | Gill | Mantle |
| 28 | 75.04 ± 0.93 | 67.49 ± 0.46 | 72.09 ± 0.76 | 87.13 ± 1.89 |
| 30 | 73.30 ± 0.72 | 70.44 ± 0.81* | 74.76 ± 1.11* | 79.40 ± 2.90* |
| 32 | 75.79 ± 1.04* | 68.81 ± 0.58* | 76.50 ± 1.20* | 73.38 ± 1.65* |
| 34 | 82.45 ± 1.22* | 69.54 ± 0.45* | 76.11 ± 0.68* | 75.64 ± 2.02* |
| 36 | 77.57 ± 0.23* | 72.53 ± 0.31* | 79.88 ± 0.30* | 82.12 ± 1.56* |
| 38 | 76.16 ± 0.54* | 73.92 ± 0.47* | 80.26 ± 0.77* | 88.45 ± 2.33* |
| 40 | 76.97 ± 0.24* | 68.36 ± 0.15 | 72.40 ± 0.04* | 76.63 ± 1.66* |

S1B Table

| HS Treatment | *Pv*Hsp70-2 (mean density ± stdev/mm^2^) | | | |
| --- | --- | --- | --- | --- |
| (^o^C) | Adductor muscle | Foot | Gill | Mantle |
| 28 | 0.00 ± 0.00 | 0.00 ± 0.00 | 0.00 ± 0.00 | 0.00 ± 0.00 |
| 30 | 0.00 ± 0.00 | 0.00 ± 0.00 | 0.00 ± 0.00 | 0.00 ± 0.00 |
| 32 | 0.00 ± 0.00 | 0.00 ± 0.00 | 0.00 ± 0.00 | 0.00 ± 0.00 |
| 34 | 0.00 ± 0.00 | 0.00 ± 0.00 | 0.00 ± 0.00 | 0.00 ± 0.00 |
| 36 | 0.00 ± 0.00 | 71.49 ± 0.29* | 74.97 ± 0.48* | 69.17 ± 0.73* |
| 38 | 75.03 ± 0.63* | 73.85 ± 0.61* | 95.68 ± 1.04* | 79.07 ± 1.51* |
| 40 | 25.00 ± 0.22* | 72.54 ± 0.27* | 85.66 ± 0.08* | 83.22 ± 2.17* |

S1C Table

| Recovery | *Pv*Hsp70-1 (mean density ± stdev/mm^2^) | | | |
| --- | --- | --- | --- | --- |
| Time | Adductor muscle | Foot | Gill | Mantle |
| C (Non-HS) | 76.27 ± 0.88 | 75.66 ± 0.58 | 73.88 ± 0.35 | 74.53 ± 0.37 |
| 0h | 79.39 ± 0.78* | 82.39 ± 1.00* | 75.70 ± 0.28* | 71.92 ± 0.23* |
| 6h | 80.22 ± 0.97* | 80.76 ± 0.13* | 78.61 ± 0.03* | 87.10 ± 0.58* |
| 12h | 81.43 ± 1.34* | 78.55 ± 0.07* | 86.50 ± 0.73* | 85.42 ± 0.25* |
| 1d | 83.70 ± 1.31* | 76.87 ± 0.27* | 83.64 ± 0.36* | 86.99 ± 0.03* |
| 2d | 85.89 ± 1.42* | 81.36 ± 0.27* | 88.15 ± 0.50* | 99.88 ± 1.39* |
| 4d | 79.19 ± 0.98* | 79.83 ± 0.18* | 87.01 ± 0.19* | 79.24 ± 0.38* |
| 6d | 81.13 ± 1.11* | 77.75 ± 0.55* | 75.29 ± 0.23 | 75.15 ± 0.20* |
| 8d | 76.08 ± 0.43 | 77.25 ± 0.19* | 81.07 ± 1.66* | 73.09 ± 0.05* |
| 10d | 73.39 ± 0.55* | 77.94 ± 0.42* | 74.43 ± 0.11* | 73.59 ± 0.09* |

S1D Table

| Recovery | *PvHsp70-2* (mean density ± stdev/mm^2^) | | | |
| --- | --- | --- | --- | --- |
| Time | Adductor muscle | Foot | Gill | Mantle |
| C (Non-HS) | 0.00 ± 0.00 | 0.00 ± 0.00 | 0.00 ± 0.00 | 0.00 ± 0.00 |
| 0h | 0.00 ± 0.00 | 0.00 ± 0.00 | 0.00 ± 0.00 | 0.00 ± 0.00 |
| 6h | 77.09 ± 0.89* | 80.09 ± 0.76* | 81.76 ± 0.69* | 75.48 ± 0.05* |
| 12h | 72.28 ± 0.62* | 84.10 ± 0.06* | 78.41 ± 0.64* | 81.28 ± 0.30* |
| 1d | 70.95 ± 0.20* | 81.90 ± 0.23* | 89.52 ± 0.61* | 80.25 ± 0.32* |
| 2d | 0.00 ± 0.00 | 79.14 ± 0.19* | 78.58 ± 0.57* | 78.63 ± 0.81* |
| 4d | 0.00 ± 0.00 | 76.82 ± 0.04* | 82.69 ± 0.15* | 76.16 ± 0.25* |
| 6d | 0.00 ± 0.00 | 74.80 ± 0.22* | 71.44 ± 0.13* | 72.14 ± 0.24* |
| 8d | 0.00 ± 0.00 | 74.29 ± 0.05* | 69.90 ± 0.23* | 69.22 ± 0.07* |
| 10d | 0.00 ± 0.00 | 75.49 ± 0.08* | 71.50 ± 0.01* | 70.28 ± 0.05* |
